# A thermophilic 8-oxoguanine DNA glycosylase from *Thermococcus barophilus*s Ch5 is a new member of AGOG DNA glycosylase family: Biochemical and functional characterization of archaeal 8oxoG DNA glycosylase

**DOI:** 10.3724/abbs.2022072

**Published:** 2022-06-13

**Authors:** Lei Wang, Donghao Jiang, Likui Zhang

**Affiliations:** 1 College of Environmental Science and Engineering Marine Science & Technology Institute Yangzhou University Yangzhou 225127 China; 2 Guangling College Yangzhou University Yangzhou 225000 China

**Keywords:** hyperthermophilic Archaea, 8-oxoguanine, 8oxoG DNA glycosylase, biochemical characteristic, base excision repair

## Abstract

8-Oxoguanine (8oxoG) in DNA is a major oxidized base that poses a severe threat to genome stability. To counteract the mutagenic effect generated by 8oxoG in DNA, cells have evolved 8oxoG DNA glycosylase (OGG) that can excise this oxidized base from DNA. Currently, OGG enzymes have been divided into three families: OGG1, OGG2 and AGOG (archaeal 8oxoG DNA glycosylase). Due to the limited reports, our understanding on AGOG enzymes remains incomplete. Herein, we present evidence that an AGOG from the hyperthermophilic euryarchaeon *Thermo Coccus barophilus* Ch5 (Tb-AGOG) excises 8oxoG from DNA at high temperature. The enzyme displays maximum efficiency at 75°C–95°C and at pH 9.0. As expected, Tb-AGOG is a bifunctional glycosylase that harbors glycosylase activity and AP (apurinic/apyrimidinic) lyase activity. Importantly, we reveal for the first time that residue D41 in Tb-AGOG is essential for 8oxoG excision and intermediate formation, but not essential for DNA binding or AP cleavage. Furthermore, residue E79 in Tb-AGOG is essential for 8oxoG excision and intermediate formation, and is partially involved in DNA binding and AP cleavage, which has not been described among the reported AGOG members to date. Overall, our work provides new insights into catalytic mechanism of AGOG enzymes.

## Introduction

8-Oxoguanine (8oxoG) is one of the major and most deleterious products of oxidized bases in DNA that arises from reactive oxygen species in vivo and oxidizing agents or ionizing radiation *in vitro* [1]. In addition to forming a canonical Watson-Crick pair with cytosine (8oxoG:C) in the anti-conformation, 8oxoG can form a stable Hoogsteen pair with adenine (8oxoG:A) in the syn-conformation, thus leading to G:C→T:A mutation when dAMP is incorporated opposite 8oxoG during replication [ 2, 3] . Additionally, A:T→C:G mutation can be generated by incorporating 8-oxodGMP that originates from dGTP oxidation opposite a template base dA by DNA polymerase [3]. Thus, 8oxoG in DNA needs to be repaired, since it is mutagenic to cells. Fortunately, cells have evolved a base excision repair process that is triggered by 8oxoG DNA glycosylase (OGG) for repairing 8oxoG in DNA. Additionally, MutY/MutYH and MutT/MTH1 enzymes are important enzymes for such DNA repair system [4].

Sequence analysis demonstrates that OGG enzymes harbor a Helix-hairpin-Helix (HhH) signature motif, belonging to the HhH superfamily [ 5, 6] . The conserved lysine in HhH motif in OGG enzymes is a key catalytic residue for catalysis and covalent intermediate formation [ 7– 10] . Thus, OGG enzymes are bifunctional glycosylases that possess glycosylase activity and AP (apurinic/apyrimidinic) lyase activity, capable of excising 8oxoG from DNA to generate an AP site and further cleave the phosphodiester bond of the created AP site [11].

Based on their amino acid similarity, OGG enzymes have been divided into three families to date: OGG1, OGG2, and archaeal 8oxoG glycosylase (AGOG) [11]. The OGG1 family members are majorly found in eukaryotes, including the well-studied human OGGs [ 12– 17] and *Saccharomyces cerevisiae* OGGs [ 5, 7, 9, 18– 21] , and in a few bacteria, such as the well-characterized *Clostridium acetobutylicum* OGGs [ 22– 24] . The OGG2 family comprises the OGG members from hyperthermophilic Archaea (HA): *Methanocaldococcus jannaschii* [25], *Sulfolobus solfataricus* [26], *Archaeoglobus fulgidus* [27], and from bacteria such as *Thermotoga maritima* [28]. The OGG1 and OGG2 family enzymes vary in sizes, base specificity and structural characteristics. The AGOG family enzymes are exclusively found in Archaea, possessing similar structures with the OGG2 family members [29]. Intriguingly, the AGOG members possess a non-canonical HhH motif [29], which is distinct from the conserved HhH motif of OGG1 and OGG2 enzymes. Currently, only three AGOG family members have been characterized, which originate from *Pyrobaculum aerophilum* [ 29– 31] , *Thermococcus gammatolerans* [ 32, 33] , and *Thermococcus kodakarensis* [34]. Their common characteristics are thermophilicity and thermostability. Among them, the *P*. *aerophilum* AGOG (Pa-AGOG) is the well-characterized AGOG [ 29– 31] . In addition, genetic data have confirmed the role of the *T*. *kodakarensis* AGOG in removing 8oxoG from DNA *in vivo* [34]. However, our understanding on AGOG function and catalytic mechanism remains incomplete due to the limited reports.

*Thermococcus barophilus* Ch5, a hyperthermophilic and piezophilic euryarchaeon that grows optimally at 85°C and 40 MPa pressure, was isolated from a deep-sea hydrothermal field of the Mid-Atlantic Ridge (Logachev field chimney, 3020-m depth) [35]. *T*. *barophilus* Ch5 is available from the UBO Culture Collection (accession number: UBOCC-M-3206). A putative AGOG protein is encoded in the genome of *T*. *barophilus* Ch5 (Tb-AGOG) [36].

In the present study, we cloned and expressed the Tb-AGOG gene, purified the gene expression product and characterized it biochemically. Our biochemical data demonstrate that Tb-AGOG can excise 8oxoG from DNA at high temperature and is quite thermostable. The enzyme is a bifunctional DNA glycosylase, harboring glycosylase activity and AP lyase activity as observed in other OGG homologues. Importantly, we dissect the roles of five conserved residues D41, E79, K163, Y174 and D229 in Tb-AGOG in excising 8oxoG from DNA, demonstrating that residues D41, E79, K163, and D229 are critical for 8oxoG removal.

## Materials and Methods

### Cloning, expression, and purification of Tb-AGOG

According to the methods described previously [32], we cloned the gene *TBCH5v1_0500* encoding the Tb-AGOG protein into the vector pET-30a (+) (Novagen, Darmstadt, Germany). The sequences of the two primers are listed in Table 1. After being verified by sequencing, the recombinant plasmid was transformed into *E*. *coli* BL21 (DE3) pLysS cells (Transgene, Beijing, China) for protein expression.

**Table:** **Table 1** Sequences of primers and oligonucleotides used in this study

Name	Sequence (5′→3′)
Tb-AGOG F	GGGAATTC *CATATG*ACCCTTGACCGCTTCATCACA
Tb-AGOG R	CCG *CTCGAG*AAGGGAAGCCAGGTCAAAGAT
D41AF	TATCGAGGAGAAAGTTG CTCTGCAGTTTTCG
D41AR	GCAACTTTCTCCTCGATAGTTCTGGCACATT
E79A F	GCTGACAGCCACTGGAG CAGAGTGGTGGTGG
E79A R	GCTCCAGTGGCTGTCAGCTGATAGCTGACTA
K163A F	ATTCCAAACGAAATGCC GCAACAATAGTCTTT
K163A R	GCGGCATTTCGTTTGGAATTCAGCACTTTTGC
Y174A F	CTGTGAAGATGTTTGGG GCTGCGGGCAGAATA
Y174A R	GCCCCAAACATCTTCACAGCAAAGACTATTGT
D229A F	TCCCACCTCTTCACATA GCTTCAATTCTCTGG
D229A R	GCTATGTGAAGAGGTGGGATACCAACGTCTCT
p45	Hex-CGAACTGCCTGGAATCCTGACGACXTGTAGCGAACGATCACCTCA
t45	TGAGGTGATCGTTCGCTACAYGTCGTCAGGATTCCAGGCAGTTCG

Restriction sites are underlined. The substitution bases are shown in italic. X: 8oxoG. Y: A, T, C or G.

The Tb-AGOG protein was expressed by adding isopropyl β-D-1-thiogalactopyranoside (IPTG), purified and stored as described for *T*. *gammatolerans* AGOG (Tg-AGOG) protein [32]. The molar extinction coefficient of Tb-AGOG protein was theoretically predicted to be 45,505 M ^−1^ cm ^−1^ on a server ( https://web.expasy.org/protparam) [37]. The Tb-AGOG protein concentration was measured by determining its absorbance at 280 nm.

### Homology modeling of Tb-AGOG

Based on the crystal structure of Pa-AGOG (PDB: 1XQP) as a template, the homology modeling of Tb-AGOG was constructed on a SWISS MODEL server ( https://swissmodel.expasy.org). By using PyMOL software (Schrodinger, LLC, https://pymol.org), we illustrated the simply-visualized mutation sites in the modeled structure of Tb-AGOG.

### Site-directed mutagenesis

By using a site-directed mutagenesis kit (Transgene), we constructed the Tb-AGOG D41A, E79A, K163A, Y174A and D229A mutant plasmids using the vector plasmid harboring the wild-type (WT) gene as a template. These mutagenic primer sequences are summarized in Table 1. After the desired mutations were verified by sequencing, we expressed and purified the Tb-AGOG mutant proteins as described for the WT protein.

### DNA glycosylase/lyase activity assays

We synthesized normal and 8oxoG-containing oligonucleotides labeled with Hex at 5′-terminus at Sangon Biotech Company (Shanghai, China). The Hex-labeled oligonucleotide sequences are summarized in Table 1. The Hex-labeled duplexes were prepared as described previously [32].

The standard DNA glycosylase reactions were performed by incubating 100 nM Tb-AGOG with 50 nM 8oxoG-containing ssDNA in the 10 μL buffer (20 mM Glycine-NaOH, pH 9.0, 8% glycerol, and 1 mM DTT) at 75 ^o^C for 1 h, unless stated otherwise. The reactions were terminated with the addition of 98% formamide on ice. The reaction products were heated at 95°C for 3 min and chilled rapidly on ice, and loaded onto a denaturing 12% polyacrylamide gel containing 8 M urea for electrophoresis. After the gels were scanned and visualized with a molecular image analyzer (PharosFx System; Bio-Rad, Hercules, USA), the DNA bands in the gels were quantified with ImageQuant software (Molecular Dynamics, Sunnyvale, USA). The detailed reaction conditions were illustrated in each figure legend. All DNA glycosylase assays were repeated three times.

### AP lyase activity assays

The AP lyase activity of Tb-AGOG was determined as described previously [38]. Briefly, we prepared the AP-containing ssDNA by incubating 0.1 μL *E*. *coli* UDG (0.1 U) (Thermo Scientific, Waltham, USA) with 100 nM uracil-containing ssDNA in the reaction buffer containing 20 mM Tris-HCl, pH 8.0, 8% glycerol and 1 mM DTT at 37 ^o^C for 15 min as described in the manual instruction. Next, we used the AP-containing ssDNA to determine the AP lyase activity of the WT/mutant Tb-AGOG protein. The AP lyase activity assays were performed by incubating 200 nM WT/mutant Tb-AGOG protein with 100 nM AP-containing ssDNA in the reaction buffer (20 mM Glycine-NaOH, pH 9.0, 8% glycerol, and 1 mM DTT) at 75°C for 1 h. The reactions were terminated and the reaction products were treated as described above. All the AP lyase assays were repeated three times.

### DNA binding assays

The DNA-binding assays of Tb-AGOG were performed in a buffer containing 20 mM Tris-HCl, pH 8.0, 100 nM Hex-labeled ssDNA, 1 mM DTT, 8% glycerol and 2000 nM WT/mutant Tb-AGOG protein at 25°C for 10 min. The bound DNA was separated by electrophoresis in a 4% native polyacrylamide gel with 0.1×TBE (Tris-borate-EDTA) buffer. The gels were visualized with a molecular image analyzer (Bio-Rad) and the DNA bands were quantified with the ImageQuant software. All DNA-binding assays were repeated three times.

### DNA trapping assays

DNA trapping assays were performed as described previously [38]. Briefly, 100 nM WT/mutant Tb-AGOG protein was incubated with 200 nM 8oxoG-containing ssDNA in a 10 μL reaction buffer (20 mM Glycine-NaOH, pH 9.0, 8% glycerol, and 1 mM DTT) in the presence of 15 mM NaBH _4_ at 75 ^o^C for 1 h. After the reactions were terminated on ice, the reaction products were heated at 100 ^o^C for 3 min, and loaded with 2.5 μL SDS-loading buffer containing 25 mM Tris-HCl, pH 6.8, 5% SDS, and 50% glycerol into a 10% SDS-PAGE. The gel was visualized and the DNA bands in the gel were quantified as described above. All DNA trapping assays were repeated three times.

## Results

### The genome of *T*. *barophilus* Ch5 encodes a putative AGOG

**Figure 1:**
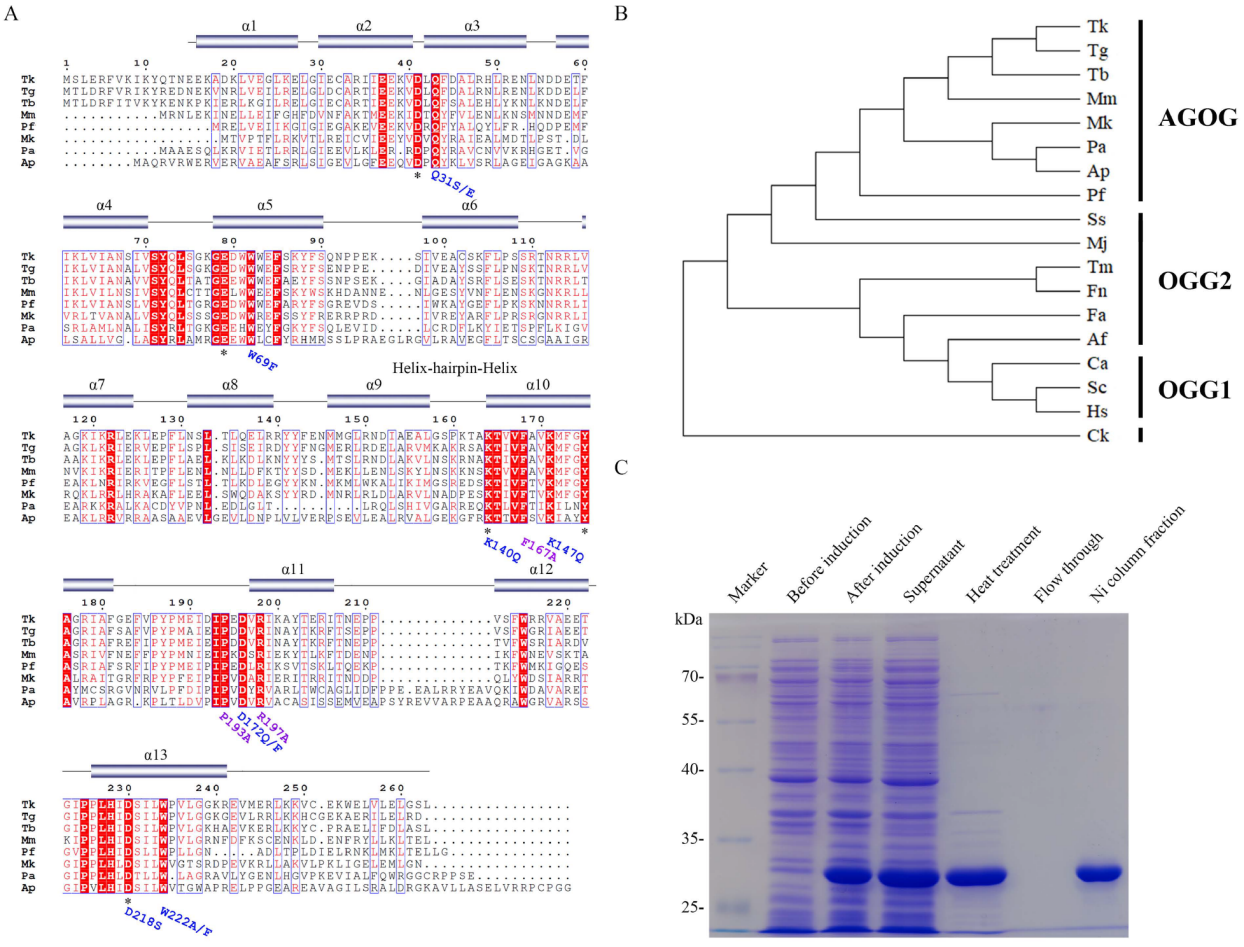
Figure 1 A putative AGOG protein is encoded in the genome of *T*. *barophilus* Ch5 (A) Sequence alignment of archaeal AGOG proteins. Tb: Thermococcus barophilus Ch5 (ALM74468); Tk: Thermococcus kodakarensis (Q5JI79); Tg: Thermococcus gammatolerans (ACS34155); Mk: Methanopyrus kandleri (Q8TXW8); Mm: Methanococcus maripaludis (Q6M0G7); Pa: Pyrobaculum aerophilum (Q8ZVK6); Ap: Aeropyrum pernix (Q9YE60); Pf: Pyrococcus furiosus (Q8U2D5). The conserved amino acid residues are boxed with shading red. The mutated amino acid residues are shown with “*”. (B) The phylogenetic tree of Tb-AGOG and other OGG enzymes. Ss: Saccharolobus solfataricus (AAK41186); Mj: Methanococcus jannaschii (Q58134); Fa: Ferroplasma acidarmanus (AGO60906); Af: Archaeoglobus fulgidus (WP_010877878); Ck: Candidatus Korarchaeum (B1L640); Tm: Thermotoga maritima (NCBI: AGL50755); Fn: Fervidobacterium nodosum (ABS60660); Ca: Clostridium acetobutylicum (KHD37022); Hs: Homo sapiens (AAB84013); and Sc: Saccharomyces cerevisiae (AAC49312). (C) Expression and purification of Tb-AGOG protein. IPTG was added to induce the Tb-AGOG gene expression. The Tb-AGOG protein was purified via sonication, heat treatment and Ni column affinity purification.

A putative AGOG protein is encoded in the genome of *T*. *barophilus* Ch5 (accession number: ALM74468). Sequence alignment shows that Tb-AGOG possesses the conserved residues and motifs in other AGOG homologues ( Figure 1A), including non-canonical HhH motif present in Pa-AGOG [29], demonstrating that the enzyme resembles other AGOG homologues and thus should excise 8oxoG from DNA. Additionally, the enzyme displays 66%, 65%, 65%, 47%, 47%, 45%, and 26% similarity with the AGOG homologues from HA: *T*. *kodakarensis*, *T*. *gammatolerans*, *M. kandleri*, *Methanococcus maripaludis*, *P*. *aerophilum*, *Aeropyrum pernix* and *Pyrococcus furiosus*, respectively. Thus, Tb-AGOG is a putative bifunctional glycosylase that harbors glycosylase activity and AP lyase activity, and should excise 8oxoG from DNA.

A complete phylogenetic analysis showed that the OGG enzymes from several archaeal species belong to the OGG2 and AGOG branches ( Figure 1B). Meanwhile, the bacterial OGG enzymes, such as *T*. *maritime* OGG and *C*. *acetobutylicum* OGG, are clustered into the OGG1 and OGG2 branches, while the eukaryotic OGG homologues are clustered into the OGG1 branch. Intriguingly, the OGG homologue from *Candidatus Korarchaeum* is in a distinct evolutionary branch that does not belong to any of the three OGG families.

To dissect the biochemical characteristics and roles of the uncharacterized conserved residues of Tb-AGOG, we cloned the enzyme gene, expressed and purified the gene product. As shown in Figure 1C, we successfully expressed the *Tb-AGOG* gene with the addition of IPTG, and purified the gene expression product with a molecular weight of ~30.6 kDa via cell sonication, heat treatment and Nickel-affinity chromatography.

### Tb-AGOG can remove 8oxoG from ssDNA and dsDNA

**Figure 2:**
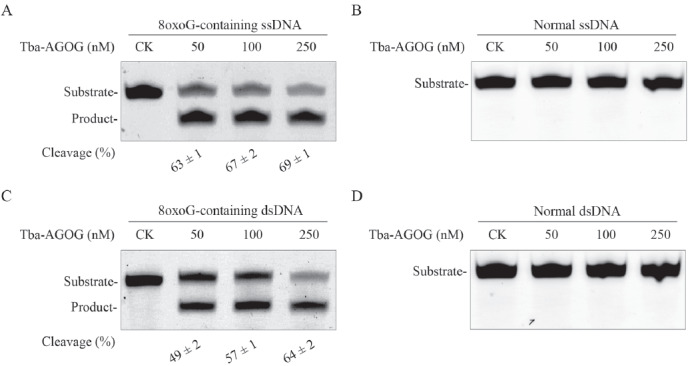
Figure 2 Tb-AGOG can excise 8oxoG from DNA DNA cleavage reactions were performed using 100 normal or 8oxoG-containing DNA as the substrate in the presence of 100 nM Tb-AGOG at 70°C for 1 h. Samples were separated by electrophoresis in a 12% denaturing gel. (A) Cleavage of 8oxoG-containing ssDNA. (B) Cleavage of normal ssDNA. (C) Cleavage of 8oxoG-containing dsDNA. (D) Cleavage of normal dsDNA. CK: the reaction without Tb-AGOG.

After purifying Tb-AGOG protein, we determined whether it can remove 8oxoG from ssDNA and dsDNA. Using the 8oxoG-containing ssDNA and 8oxoG:C-containing dsDNA as the substrates, we performed DNA cleavage reactions of Tb-AGOG at 65 ^o^C. As shown in Figure 2A, we observed the cleaved product at the tested enzyme concentrations when using 8oxoG-containing ssDNA as the substrate. By contrast, no cleaved product was observed when normal ssDNA was used as the substrate ( Figure 2B). The enzyme displayed similar efficiency in cleaving 8oxoG:C-containing dsDNA compared with that in cleaving 8oxoG-containing ssDNA, ( Figure 2C). Likewise, Tb-AGOG is inactive to cleaving normal dsDNA ( Figure 2D). Overall, Tb-AGOG can excise 8oxoG from ssDNA and dsDNA with similar efficiency at high temperature.

### Biochemical characteristics of Tb-AGOG

**Figure 3:**
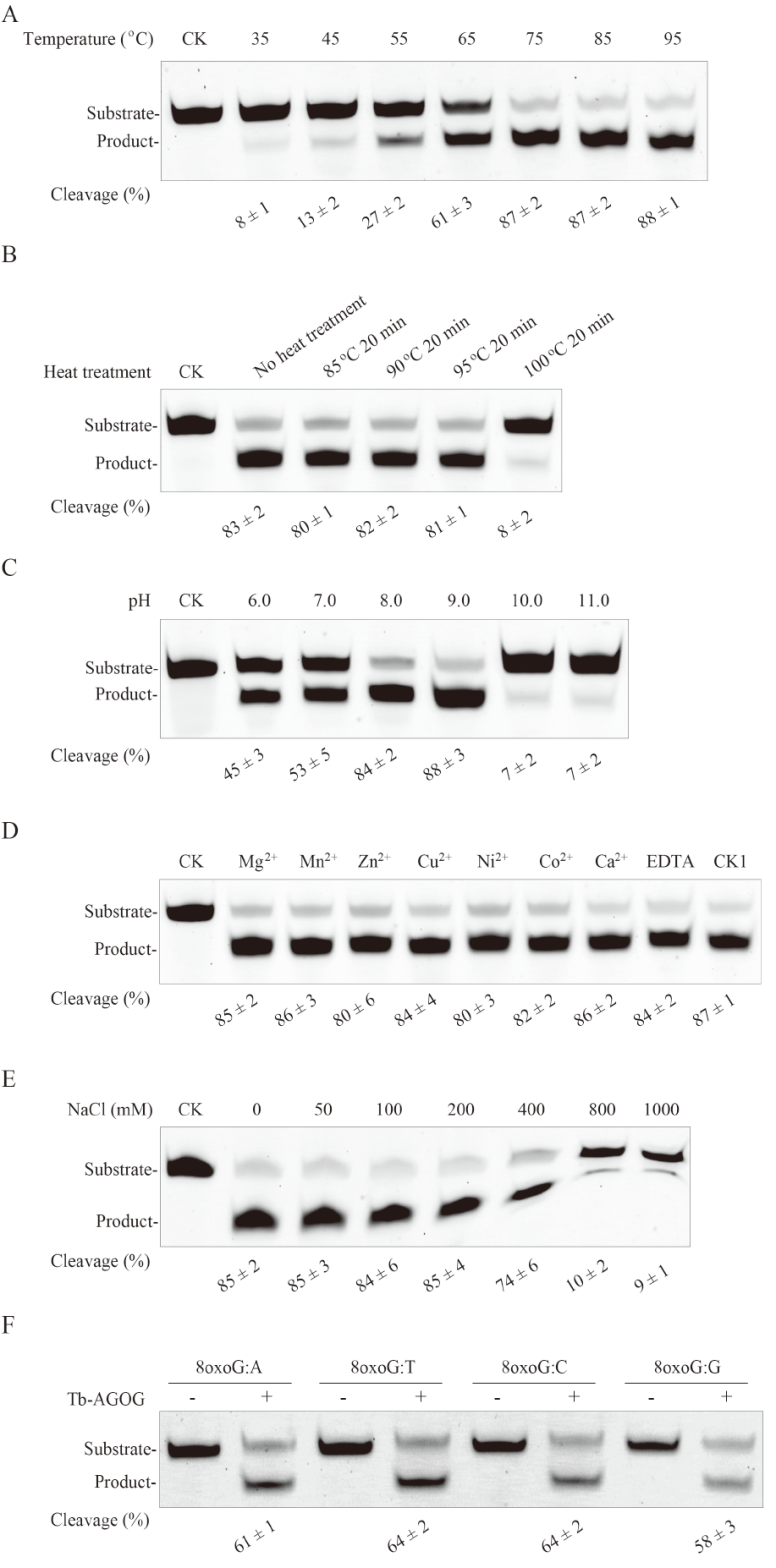
Figure 3 Biochemical characteristics of Tb-AGOG DNA cleavage reactions were performed with Tb-AGOG using the 8oxoG-containing ssDNA as the substrate under various conditions to investigate its biochemical characteristics. (A) The optimal temperature assays of the Tb-AGOG activity. (B) The thermo-tolerance assays of Tb-AGOG. (C) The optimal pH assays of the Tb-AGOG activity. (D) Effects of various divalent cations on the Tb-AGOG activity. (E) Effect of NaCl on the Tb-AGOG activity. (F) Substrate specificity of Tb-AGOG. DNA cleavage reactions were performed with Tb-AGOG using the 8oxoG-containing dsDNA as the substrate at 70 oC and pH 8.0. CK in panels A, B, C, E and F, and CK1 in panel B: the reaction without Tb-AGOG; CK1 in panel D: the reaction without divalent cations.

We employed 8oxoG-containing ssDNA as the substrate to determine the biochemical characteristics of Tb-AGOG protein. First, we investigated the optimal temperature of the enzyme by performing DNA cleavage reactions at various temperatures ranging from 35 ^o^C to 95 ^o^C. As shown in Figure 3A, the 8oxoG-conatining ssDNA substrate was cleaved by Tb-AGOG with varied efficiencies as the reaction temperature was increased from 35 ^o^C to 95 ^o^C. Specifically, Tb-AGOG displayed maximum efficiency at 75 ^o^C–95 ^o^C, demonstrating that the enzyme has the optimal reaction temperature of 75 ^o^C–95 ^o^C. We further found that after 20 min of heating at 100 ^o^C, the heated Tb-AGOG protein only retained 8% cleavage activity ( Figure 3B), suggesting that the enzyme abolishes most of the activity after heat treatment at 100 ^o^C.

Meanwhile, maximum cleavage efficiency of Tb-AGOG was observed at pH 9.0 ( Figure 3C), suggesting that the optimal reaction pH of the enzyme activity is 9.0. Furthermore, we found that Tb-AGOG is independent of divalent metal ions for excising 8oxoG from DNA, since the enzyme exhibits similar cleavage efficiency in the presence of 10 mM EDTA or 2 mM of other divalent metal ions ( Figure 3D). Additionally, we observed that NaCl is not required for Tb-AGOG to excise 8oxoG from DNA ( Figure 3E), and the enzyme activity can be inhibited by high concentration of NaCl. Finally, we investigated substrate specificity of Tb-AGOG using 8oxoG-containing dsDNA with four different mispairs as substrates, and demonstrated that the enzyme displays similar efficiency for cleaving 8oxoG:A-, 8oxoG:T-, 8oxoG:C- or 8oxoG:G-containing dsDNA ( Figure 3F).

### Structural modeling of Tb-AGOG

**Figure 4:**
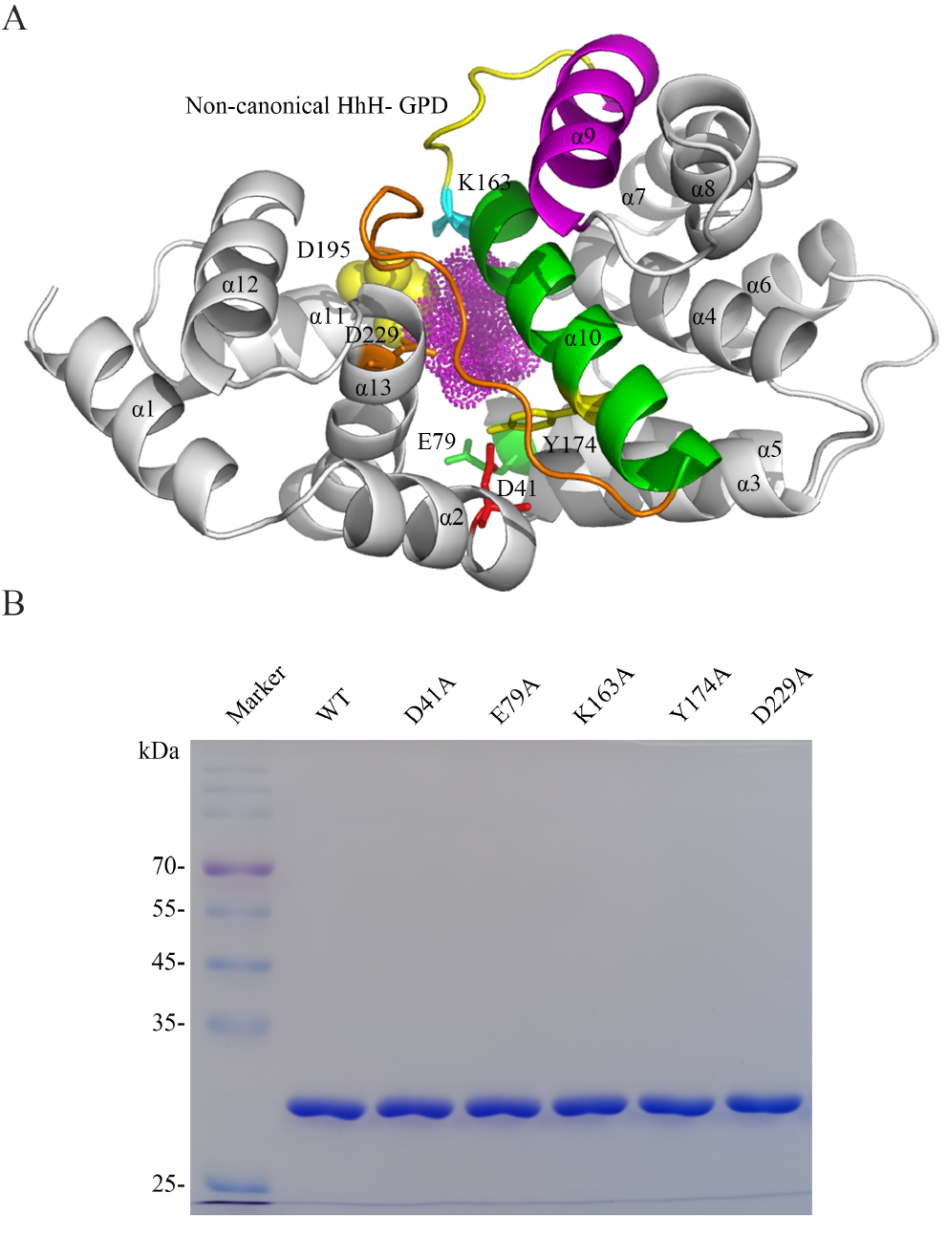
Figure 4 Homology modeling of Tb-AGOG and purification of the mutant proteins (A) The modeled structure of Tb-AGOG protein. The non-canonical HhH motif is colored with magentas and green. The conserved residue D195 in the GPD motif is shown with balls. The mutated residues D41, E79, K163, Y174 and D229 in Tb-AGOG are shown with sticks. (B) Purification of the Tb-AGOG mutant proteins.

Currently, the crystal structures of Pa-AGOG protein and the AGOG from *P*. furiosus (1XG7, 4PII) have been solved among the reported AGOG homologues [29]. Although the roles of the several conserved residues in Pa-AGOG have been dissected [31], the functions of lots of conserved residues in AGOG homologues remain unclear. To dissect the structural and functional relationship of Tb-AGOG, we constructed its structure homology model using the crystal structure of Pa-AGOG as a template. As shown in Figure 4A, Tb-AGOG harbors 13 α-helices, possessing a non-canonical HhH structure as demonstrated in Pa-AGOG [29]. The residue K163 in the non-canonical HhH motif of Tb-AGOG is conserved in other bifunctional glycosylases, suggesting that this residue should be essential for catalysis. Additionally, 8oxoG is encircled with the conserved residues D41 that is located between α2 and α3, E79 in α5, Y174 in α10, and D229 in α13 ( Figures 1 and 4 ). These residues are highly conserved in other AGOG members, which prompted us to propose that they might play important roles in 8oxoG recognition and removal. To investigate the roles of these conserved residues in the enzyme in removing 8oxoG from DNA, we constructed the Tb-AGOG D41A, E79A, K163A, Y174A, and D229A mutants by site-directed mutagenesis. According to the procedures as described for the WT protein, we successfully purified these mutant proteins ( Figure 4B).

### Cleavage of 8oxoG-containing ssDNA by the Tb-AGOG mutants

**Figure 5:**
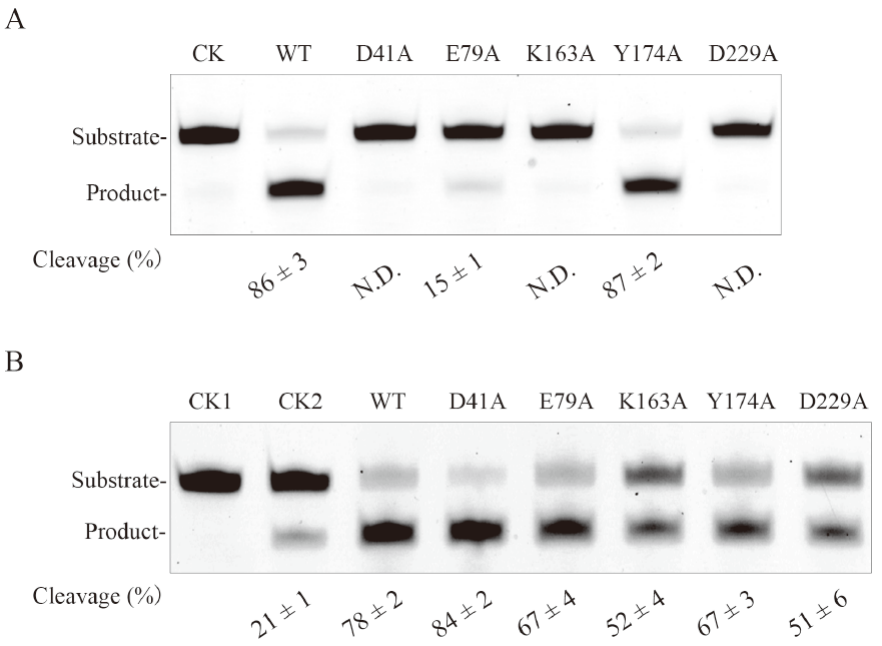
Figure 5 Cleavage of 8oxoG- and AP-containing ssDNA by the WT/mutant Tb-AGOG protein The 8oxoG-containing ssDNA (100 nM) was incubated with 100 nM WT/mutant Tb-AGOG protein at 70 oC for 1 h. Samples were separated by electrophoresis in a 12% denaturing gel. (A) The 8oxoG-containing ssDNA cleavage by the WT/mutant Tb-AGOG protein. (B) The AP-containing ssDNA cleavage by the WT/mutant Tb-AGOG protein. CK in the panel A: the reaction without Tb-AGOG. CK1 in the panel B: only 8oxoG-containing ssDNA. CK2 in the panel B: the reaction without Tb-AGOG.

After purifying the Tb-AGOG mutant proteins, we determined their efficiencies for removing 8oxoG from DNA using 8oxoG-containing ssDNA as the substrate. As shown in Figure 5A, we found that the D41A, K163A and D229A substitutions almost completely abolished the cleavage activity of the enzyme. However, the Y174A mutant retained the activity of the WT enzyme, which indicates that the mutation of Y174 to alanine has a marginal effect on the enzyme activity. Additionally, the E79A mutant displayed the reduced cleavage efficiency (15%), compared with the WT protein, suggesting that the substitution of E79 with alanine causes the compromised activity. Thus, these observations suggest that residues D41, K163 and D229 in Tb-AGOG are essential for excising 8oxoG from DNA, residue E79 is partially responsible for catalysis, but residue Y174 is not essential for catalysis.

### AP lyase activity of the Tb-AGOG mutants

As observed in other AGOG homologues [31], Tb-AGOG is a bifunctional glycosylase, harboring the concerted AP lyase activity in addition to its glycosylase activity, since the cleaved product of the enzyme appears without treatment with hot NaOH solution. Thus, we determined whether the enzyme can act on AP-containing DNA. We used the AP-containing ssDNA created by removal of uracil from ssDNA by *E*. *coli* UDG as substrate to perform DNA cleavage reactions of Tb-AGOG at 75 ^o^C. As shown in Figure 5B, we observed 21% cleavage in the absence of Tb-AGOG, demonstrating that the spontaneous cleavage is due to the incubation of 75 ^o^C for 1 h. Compared with the control reaction without the enzyme, the WT protein displayed the increased cleavage percentage (78%), thereby confirming that the enzyme can cleave the AP-containing DNA substrate.

To explore the contributions of these five interested residues of the enzyme to the AP lyase activity, we also performed the AP-containing ssDNA cleavage assays of the Tb-AGOG mutant proteins. Compared with the WT protein, the D41A mutant displayed similar AP cleavage percentage ( Figure 5B), thereby suggesting that the mutation of D41 to alanine has no significant effect on AP lyase activity of the enzyme. By contrast, the E79A, K163A, Y174A and D229A mutants had the reduced AP cleavage activity with various degrees, which indicates that residues E79, K163, Y174 and D229 are involved in cleaving AP site.

### DNA binding of the Tb-AGOG mutants

**Figure 6:**
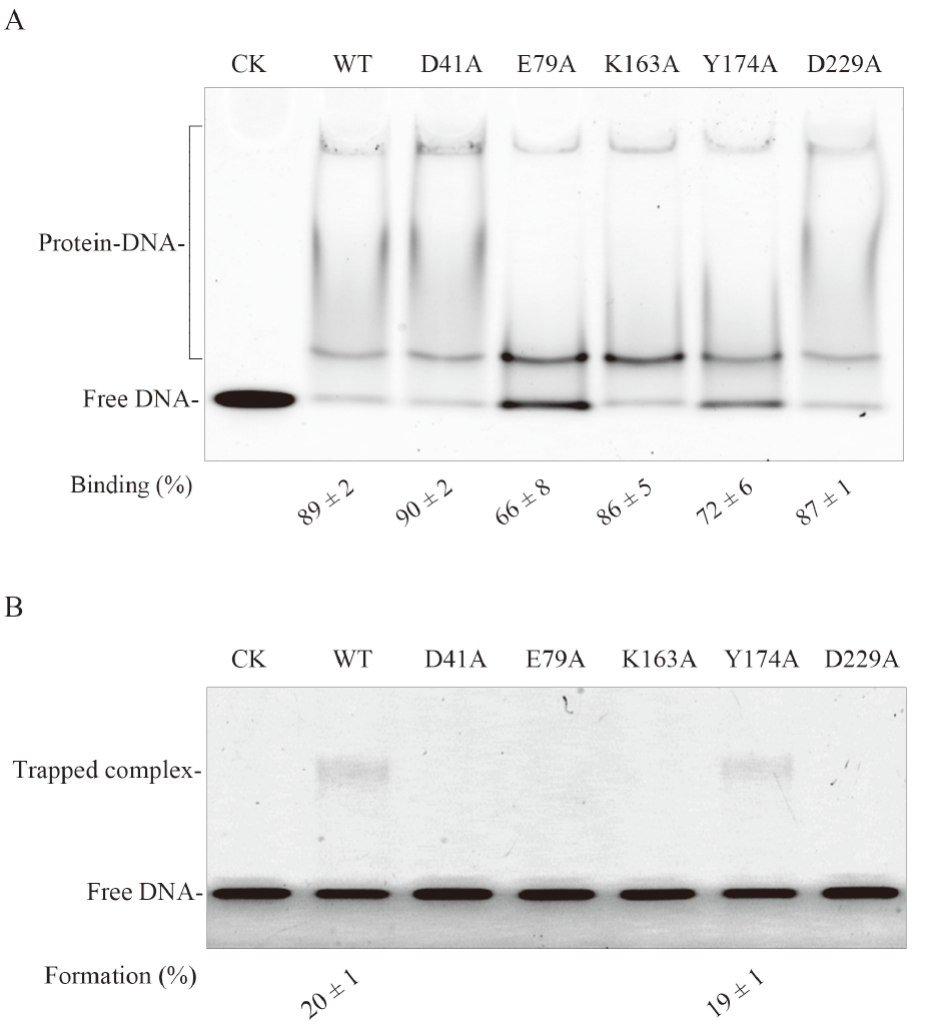
Figure 6 DNA binding and DNA trapping assays of 8oxoG-containing ssDNA of the WT/mutant Tb-AGOG protein (A) The 8oxoG-containing ssDNA binding assays of the WT/mutant Tb-AGOG protein. 100 nM 8oxoG-containing ssDNA was incubated with 2000 nM WT/mutant Tb-AGOG protein at room temperature for 10 min. Samples were separated by electrophoresis in a 4% native gel. CK: The binding without Tb-AGOG. (B) The 8oxoG-containing ssDNA trapping assays of the WT/mutant Tb-AGOG protein. 200 nM 8oxoG-containing ssDNA was incubated with 100 nM WT/mutant Tb-AGOG protein in the presence of 15 mM NaBH 4 at 75 oC for 1 h. The trapped complex was separated by electrophoresis in a 10% SDS-PAGE. CK: the reaction without Tb-AGOG.

Next, we investigated whether Tb-AGOG can bind with DNA using 8oxoG-containing ssDNA as substrate. As shown in Figure 6A, we found that Tb-AGOG can bind with 8oxoG-containing ssDNA substrate. Additionally, the enzyme displayed similar normal ssDNA binding when ssDNA without 8oxoG was used as the substrate (data not shown).

In addition, we tested the binding efficiencies of the Tb-AGOG mutants using the 8oxoG-containing ssDNA as substrate. Compared with the WT protein, the E79A and Y174A mutants displayed decreased binding with DNA, suggesting that residues E79 and Y174 are responsible for DNA binding. By contrast, the D41A, K163A and D229A mutants exhibited the comparable DNA binding to the WT protein, which suggests that residues D41, K163, and D229 are not essential for DNA binding.

### Intermediate formation of the Tb-AGOG mutants

Tb-AGOG possesses the non-canonical HhH motif that contains the conserved lysine (K163) in other HhH-containing proteins. The conserved lysine in *S*. *cerevisiae* OGG is essential for forming covalent intermediate [7], which promoted us to investigate whether a covalent intermediate can be formed between Tb-AGOG and 8oxoG-containing DNA. As shown in Figure 6B, the trapped complex between Tb-AGOG and 8oxoG-containing ssDNA was formed, suggesting that Tb-AGOG can form a covalent intermediate with 8oxoG-containing ssDNA.

Next, we examined the intermediate formation efficiencies of the Tb-AGOG mutants by performing the same DNA trapping assays as described for the WT protein. Compared with the WT protein, the D41A, E79A, K163A and D229A mutants could not form the intermediate, which suggests that residues D41, E79, K163 and D229 are essential for intermediate formation. By contrast, the Y174A mutant displayed similar intermediate formation activity to the WT protein, thereby demonstrating that the residue Y174 plays no role in forming covalent intermediates.

## Discussion

In the present study, we presented evidence that the thermophilic AGOG from the hyperthermophilic euryarchaeon *T*. *barophilus* Ch5 can remove 8oxoG from DNA at high temperature, thus confirming that all AGOG homologues harbor conserved function, since they have evolved from a common ancestor. Intriguingly, Tb-AGOG displays several biochemical characteristics distinct from other AGOG homologues. Additionally, we dissected for the first time the roles of five conserved residues in Tb-AGOG in removal of 8oxoG from DNA, which have not been described to date, thus providing new insights into the catalytic mechanism of archaeal AGOG.

Hyperthermophilic Archaea thrive at high temperatures above 80°C, thus facing an increased threat to genome stability, since the rates of spontaneous oxidation are accelerated at elevated temperatures [ 39– 41] , in addition to the increased deamination rates [42]. Surprisingly, despite living in inhospitable high-temperature environments, HA display spontaneous mutation rates similar to *E*. *coli* [ 43, 44] , thereby suggesting that they are more efficient than *E*. *coli* in repairing damaged DNA, even including DNA oxidation. Interestingly, the OGG enzymes from HA are grouped into OGG2 and AGOG families ( Figure 1B). We compared biochemical characteristics of Tb *-*AGOG with other reported OGG enzymes from Archaea ( Table 2), demonstrating that they have distinct biochemical characteristics. Firstly, the optimal reaction temperature (75 ^o^C–95 ^o^C) of Tb-AGOG is similar to that of Tg-AGOG, but clearly higher than that of *Thermoplasma volcanium* OGG (Tv-OGG) and *A*. *fulgidus* OGG (Af-OGG) [ 27, 45] . Besides, the optimal reaction pH 9.0 of Tb-AGOG is higher than that of Tg-AGOG, Tv-, Mj- and Af-OGG enzymes, respectively [ 25, 27, 32, 45] . Although these two enzymes possess high amino acid similarity, Tb-AGOG displays thermostabilty distinct from Tg-AGOG. Additionally, 100 mM NaCl is optimal for Af-OGG to remove 8oxoG from DNA [27]. By comparison, NaCl is not required for Tb-AGOG and Tg-AGOG to excise 8oxoG from DNA, and their activities are inhibited by high concentration of NaCl [32]. However, Tb-AGOG is different from Tg-AGOG in NaCl tolerance.

**Table:** **Table 2** Biochemical characteristics of Tb-AGOG and OGG homologues from other HAs

	Family	Optimal temperature ( ^o^C)	Optimal pH	Thermostabilty	Substrate specificity	NaCl effect	Reference
Tb-AGOG	AGOG	75–95	9.0	Retaining 8% activity after heated at 100 ^o^C for 20 min	Similar efficiency for 8oxoG:N	Retaining 74% activity at 400 mM NaCl	This work
Tg-AGOG	AGOG	95	7.0–8.0	Retaining 40% activity after heated at 90 ^o^C for 5 min	8oxoG:C≈8oxoG:T>8oxoG:A>8oxoG:G	No activity at > 200 mM NaCl	[32]
Af-AGOG	OGG2	60	8.5	N.D.	8oxoG:C>8oxoG:G>8oxoG:T>8oxoG:A	100 mM optimal	[27]
Tv-AGOG	OGG2	60	7.5	N.D.	8oxoG:T>8oxoG:C>8oxoG:G>8oxoG:A	N.D.	[45]
Mj-AGOG	OGG2	N.D.	8.5	N.D.	8oxoG:C≈8oxoG:T>8oxoG:A>8oxoG:G	N.D.	[25]

N.D.: Not determined.

In addition, Tb-AGOG displays similar efficiency for cleaving 8oxoG:N (A, T, C or G) as observed in *M*. *jannaschii* OGG (Mj-OGG), which is distinct from the observations for Tg-AGOG, Pa-AGOG, Tv-OGG, and Af-OGG ( Table 2). In addition to OGG enzymes, bacteria and eukarya also encode a MutY protein which is capable of excising adenine in 8oxoG:A mismatch that are formed during replication, suggesting that the MutY and OGG enzymes are complementary in their activities as part of the 8oxoG system of the base excision repair pathway [ 46– 48] . Like bacteria and eukarya, HA also encodes the bacterial MutY homologues that excise adenine from a 8oxoG:A mismatch, even including *T*. *barophilus* Ch5, which indicates that the MutY homologues in HA may be involved in 8oxoG repair. In combination with the role of the MutY homologue in this archaeon, effective cleavage of 8oxoG from a 8oxoG:A mismatch by Tb-AGOG may allow the cells to rapidly repair 8oxoG prior to replication, thus counteracting mispair by DNA polymerase. However, if the 8oxoG in the DNA arises from the oxidation of a G in a G:C basepair (to make 8oxoG:C) followed by mispairing by DNA polymerase (to make 8oxoG:A), then removing the 8oxoG by OGG will be pro-mutagenic (the adenine should be removed by MutY instead).

Residue K163 in Tb-AGOG is in the non-canonical HhH motif, which is found in Pa-AGOG [29]. As reported previously, the conserved lysine in bifunctional DNA glycosylases in Pa-AGOG is a catalytic residue, since the substitution of K140 with Q leads to the loss of activity [30]. In this study, we provide data that support this conclusion, since the substitution of K163 with alanine leads to the loss of 8oxoG removal activity and defect in the covalent intermediate formation. By comparison, the K163A mutation reduced AP cleavage (53% relative to the WT protein; Table 3), thereby suggesting that this residue is partially involved in cleaving AP site. Overall, this work improves our understanding of the function of the conserved lysine residue in the AGOG enzymes.

**Table:** **Table 3** Relative activity of the Tb-AGOG mutants

	WT ^a^	D41A	E79A	K163A	Y174A	D229A
8oxoG excision	100	N.D.	17	N.D.	100	N.D.
AP cleavage	100	107	75	53	75	52
DNA binding	100	101	74	97	81	98
Intermediate formation	100	N.D.	N.D.	N.D.	97	N.D.

^a^The activity of the WT protein was designated as 100. N.D.: Not detected.

In addition to the conserved lysine in HhH superfamily, the conserved residue D218 in α-helix 13 in Pa-AGOG is another catalytic residue, since the mutation of residue D218 to serine leads to the activity loss [31]. Like the residue D218, the residue D229 in Tb-AGOG, which is analogous to the residue D218 in Pa-AGOG, is essential for 8oxoG removal. Importantly, we first revealed the essential role of the conserved residue D229 in the covalent intermediate formation, which has not been described in the D218 in Pa-AGOG.

Residues D41, E79, and Y174 in Tb-AGOG are conserved in AGOG homologues ( Figure 1A). In this study, we explored the roles of the conserved residues D41, E79, and Y174, and demonstrated that the D41A substitution abolished 8oxoG excision and intermediate formation activity, but had no effect on DNA binding and AP cleavage ( Table 3). Additionally, the E79A mutant lacks the intermediate formation activity, and displays reduced GO excision, AP cleavage and DNA binding ( Table 3). Furthermore, the Y174A mutant has the WT 8oxoG excision activity and intermediate formation activity, with slightly reduced AP cleavage and DNA binding ( Table 3). Therefore, our mutational experiments provide new insights into catalytic mechanism of AGOG.

**Figure 7:**
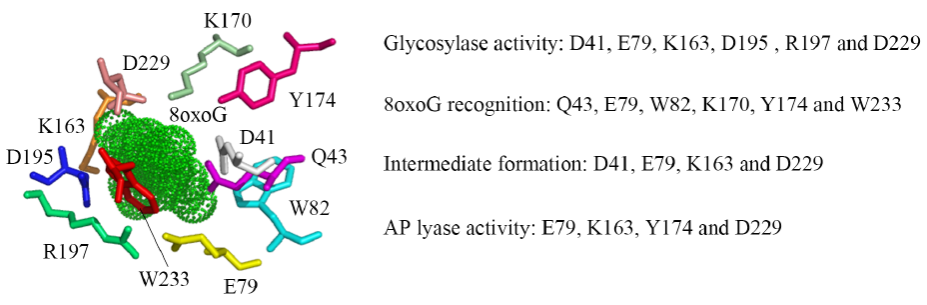
Figure 7 Proposed roles of the conserved residues in Tb-AGOG in removing 8oxoG from DNA The figure was modified from the structural model of Tb-AGOG by Pymol. 8oxoG is shown with green dots and the residues that wrap 8oxoG are labeled with sticks with different colors.

Pa-AGOG is a structurally and biochemically characterized AGOG, demonstrating that residues Q31, W69, K147, and W222 play essential roles in 8oxoG recognition, and that residues K140, D172, and D218 are essential for 8oxoG removal [31]. Meanwhile, the conserved residues F167, P193 and R197 in Tg-AGOG are not essential for 8oxoG recognition, but residue R197 is partially involved in 8oxoG excision [32]. Note that residues Q43, W82, K170, and W233 in Tb-AGOG are analogous to residues Q31, W69, K147, and W222 in Pa-AGOG, and R197 in Tb-AGOG corresponds to R197 in Tg-AGOG. Based on previous structural and mutation data and the data of this study, we proposed a reaction mechanism for removing 8oxoG from DNA by Tb-AGOG ( Figure 7): residues D41, E79, K163, D195, R197 and D229 are responsible for 8oxoG excision, residues Q43, E79, W82, K170, Y174 and W233 for 8oxoG recognition, residues D41, E79, K163 and D229 for intermediate formation, and residues E79, K163, Y174 and D229 for AP cleavage. This catalytic mechanism of Tb-AGOG may be conserved in other AGOG proteins.

In summary, we present biochemical and functional data of the thermostable AGOG from the hyperthermophilic euryarchaeon *T*. *barophilus* Ch5. Tb-AGOG can remove 8oxoG from DNA at high temperature, with maximum efficiency at 75 ^o^C–95 ^o^C and at pH 9.0. In addition, Tb-AGOG is a bifunctional DNA glycosylase that harbors glycosylase activity and AP lyase activity, as observed in other OGG homologues. Importantly, we reveal for the first time that residues D41 and D229 in Tb-AGOG, which are conserved in other AGOG family members, are essential for catalysis, in addition to the conserved residue K163. Residue E79 is partially responsible for 8oxoG removal, DNA binding, AP cleavage and intermediate formation, and residue Y174 is partially involved in DNA binding and AP cleavage. Overall, our work provides insights into the catalytic mechanism of AGOG.
